# Hip–Spine Syndrome: A Focus on the Pelvic Incidence in Hip Disorders

**DOI:** 10.3390/jcm12052034

**Published:** 2023-03-03

**Authors:** Tadatsugu Morimoto, Takaomi Kobayashi, Masatsugu Tsukamoto, Hirohito Hirata, Tomohito Yoshihara, Yu Toda, Masaaki Mawatari

**Affiliations:** Department of Orthopedic Surgery, Faculty of Medicine, Saga University, 5-1-1 Nabeshima, Saga 849-8501, Japan

**Keywords:** pelvic incidence, hip osteoarthritis, hip–spine syndrome, systematic review, sagittal spinopelvic parameters, rapidly destructive coxarthrosis, femoroacetabular impingement, femoral head osteonecrosis, development of dysplasia of the hip, osteonecrosis of the femoral head

## Abstract

Since Offierski and MacNab reported a close association between the hip and spine as hip–spine syndrome in 1983, many studies on spinal alignment in hip disorders have been conducted. Notably, the pelvic incidence angle (PI) is the most important parameter and is determined by the anatomical variations in the sacroiliac joint and hip. Studies on the association of the PI with hip disorders can help in understanding the pathophysiology of hip–spine syndrome. A PI increase has been observed during the evolution of bipedal locomotion in humans and in the acquisition of gait during child development. Although the PI is a fixed parameter that is stable and unaffected by posture from adulthood onwards, it has become clear that it increases in the standing position in older people. While it may be associated with a greater risk of developing or progressing to spinal disorders, the association between the PI and hip disorders remains controversial because of the multifactorial nature of hip osteoarthritis (HOA) and the wide range of PIs in HOA (18–96°), making the interpretation of results difficult. However, several hip disorders (i.e., femoroacetabular impingement and rapid destructive coxarthrosis) have been shown to be associated with the PI. Further investigation on this topic is, therefore, warranted.

## 1. Introduction

Hip–spine syndrome was first reported by Offierski et al. in 1983 [1]. Hip–spine syndrome was divided into four categories (i.e., simple, secondary, complex, and misdiagnosed), noting that there is a close association between the spine and hip joint that can alter their mutual alignment and that there have been many cases of overlapping conditions and misdiagnoses. In recent years, spinopelvic sagittal alignment has received much attention, and many studies have been conducted on spinal alignment in hip disorders [2]. The Scoliosis Research Society–Schwab classification for spinal deformity, including the sagittal spinopelvic parameters, has been validated for clinical correlation and treatment indications and is widely used throughout the world [2,3]. In particular, the pelvic incidence (PI), defined as the angle between a line perpendicular to the sacral plate and one connecting the midpoint of the sacral plate and the center of the femoral head, is a crucial anatomical parameter that contributes to the balance of the sagittal plane of the spine [2,3,4,5,6,7]. As can be inferred from the fact that the measurement points of the PI consist of the spine and hip joint, it is an indicator of the close relationship between the spine and hip joint. However, several studies have shown that an increased PI is associated with a higher risk of spinal disorders, such as lumbar spondylosis, spinal canal stenosis, disc degeneration, disc herniation, and facet joint arthritis [3,8,9]. On the contrary, the close association between the PI and the spine, pelvis, and hip joint demonstrates that the PI is significantly correlated with lumbar lordosis (LL) and the acetabular cup orientation [5]. Thus, the pronounced involvement of the PI in both spine and hip diseases helps in understanding the pathophysiology and mechanisms of hip–spine syndrome, a condition in which both hip and spine disorders should be considered, and in determining treatment strategies. In this review, Section 2 discusses the PI in terms of phylogeny, ontogeny, and aging. The PI gradually increases with advancing proficiency in bipedal locomotion across the human phylogeny [10,11]. Schlosser et al. [10] speculated that the increase in the PI across the human phylogeny and during ontogenesis was a necessary adaptation for acquiring a pelvis capable of supporting the trunk directly above the body while achieving energy-efficient bipedal locomotion. Considering how the spine, pelvis, and hip originally emerged from a basic ancestral body plan, one might identify the key to a deeper understanding of the PI to be rooted in vertebrate history and the pathogenesis of spinal and hip diseases. Section 3 summarizes the three factors of the PI increase. The three patterns of the PI increase are easier to understand if the PI changes during the evolutionary process are understood. Section 4 describes the relationship between the PI, lumbar disorder, and sacral morphology. Finally, Section 5 provides a summary of the relationship between hip disease and the PI. Since the role of the PI in hip disease has been less well studied than in spinal disease [12], this study focuses more on the relationship between hip disease and the PI than on spinal disease.

Compared to a systematic review, this study employs a narrative review approach that allows for a more extensive, flexible, and comprehensive organization and analysis of the existing literature on the PI, a key anatomical spinopelvic parameter. Therefore, we have selected important articles published in peer-reviewed scientific journals, specifically focusing on the PI, with the aim of providing evidence on the role of the PI in the pathogenesis of hip–spine syndrome.

## 2. PI Changes in Phylogeny, Ontogeny, and Aging

An increased PI has been observed in the transition of apes from quadrupedal walking to occasional bipedal walking and finally to permanent bipedal walking [10,11]. From Australopithecus to Homo, as the frequency of upright and bipedal walking increased and became more permanent, the PI and the associated curvature of the lumbar vertebrae and spine are thought to have increased.

**Figure 1:**
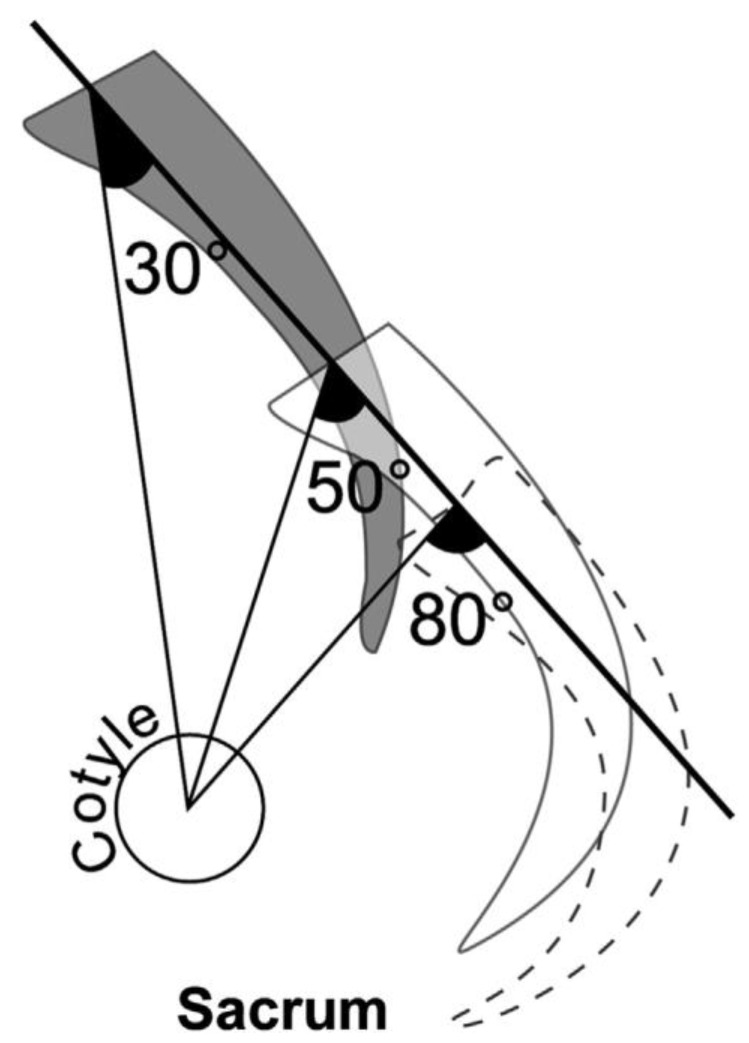
Sagittal model of pelvic incidence. Three schematic steps are depicted: a theoretical ancestral stage (30°), an australopithecine stage (50°), and an extreme range for humans (80°). Adapted from ref. [11] with permission from Elsevier.

The increase in the PI values during this evolutionary process suggests that the locomotor mode of bipedal walking may have had a significant effect on pelvic morphology. Interestingly, Hayama et al. [13] noted a significant increase in the PI as well as LL in Japanese macaques trained to walk on two legs as infants. Tardieu et al. [11] demonstrated that the vertical pelvic offset (the shortest vertical distance between the center of the femoral head and the center of the sacral endplate) decreased with increasing proficiency in bipedal locomotion across the human phylogeny. In geometry, if the vertical pelvic offset decreases, the PI increases (Figure 1) [8].

The PI increase has been observed in the evolution of bipedal locomotion in human evolution and in the acquisition of gait during child development. During human ontogenesis, the PI increases from 4 to 18 years of age, when the spine adapts to bipedal locomotion, and is considered the only fixed parameter that is stable and unaffected by posture from adulthood onwards [9,10].

The theory of a “fixed PI” assumes that there is scant movement of the sacroiliac joint (SIJ). However, from a biomechanical point of view, the mobility of the SIJ has been proven to have a nutation/counternutation motion. Nutation means that the sacrum is tilted with respect to the ilium, which increases the PI, whereas counternutation is the opposite movement, which decreases the PI [8]. The mobility of the SIJ suggests that the PI varies with posture in people with large sacroiliac joint mobility and instability, such as in those with aging-degenerated sacroiliac joints. In recent years, the concept that the PI is a fixed value has been called into question. It has been reported that the PI increases with age [14], that SIJ mobility is greater in patients with lumbar spine degeneration than in normal subjects [15], and that the PI decreases after corrective lumbar spine surgery [16,17,18,19,20]. In addition, postural changes in the PI have been reported to decrease from flexion to extension [21] and from standing to prone and supine positions [22,23]. Using CT-generated digitally reconstructed X-ray images and a slot-scan 3D X-ray imager (EOS), Hasegawa et al. [22] showed a significant difference in the PI between the prone and upright positions in individual patients. In addition, Ohya et al. [24] reported that 12.5% of patients undergoing lumbar spine surgery had a PI change of more than 10 degrees on the operating table compared to their standing PI. Therefore, PI investigation in HOA should consider age and position in the evaluation. Since the HOA study population is commonly middle-aged and older and since the PI is usually measured in the standing position, the PI may be elevated.

These facts indicate that the PI is increased in the standing position in the aging population, probably due to the instability caused by sacroiliac joint degeneration, which should be noted in pathological evaluations and surgical strategy decisions. Furthermore, in spine surgery, the correction of lumbar lordosis according to the PI has become an important component of spine surgical planning in recent years [3]. The correction of lumbar lordosis based on an apparent increased PI in the standing position may be suboptimal; in other words, overcorrection and the target modifier for the correction of lumbar lordosis in cases with large PIs are subjects for future studies.

## 3. Three Factors of the PI Increase

Tardieu et al. [11] illustrated that evolutionary changes in pelvic morphology increased the PI as the vertical distance between the sacral midpoint, the reference point for the PI, and the center of the femoral head became closer (Figure 1).

**Figure 2:**
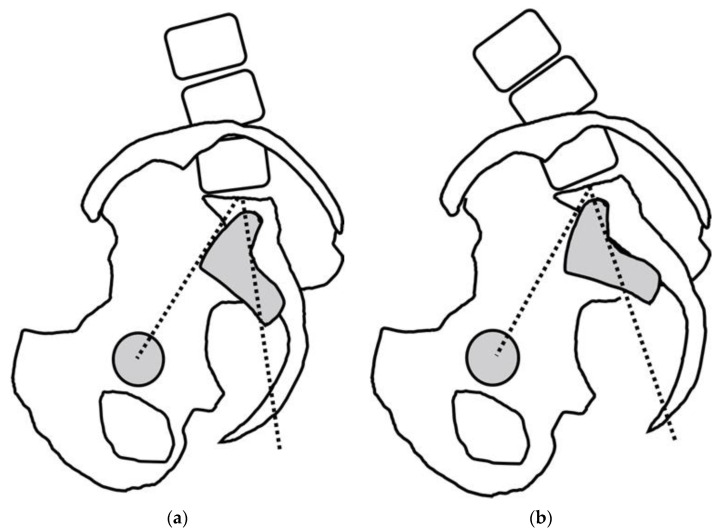
“Increasing sacroiliac joint angulation” associated with lumbar kyphosis, posterior pelvic tilt, and sacroiliac joint degeneration. (**a**) Pelvis tilted posteriorly with lumbar kyphosis. (**b**) The trunk tilts forward, and the sacrum tilts forward, “increasing the angle of the sacroiliac joint” and resulting in a higher PI. Reference [26] are cited and modified with permission from the editorial office of the Journal of Spine research.

Ramchandran et al. [8] reported three factors for the increased PI: (1) an increased horizontal offset—the distance between the center of the femoral head and the center of the sacral endplate in the anteroposterior direction, (2) a decreased vertical pelvic offset—the distance between the center of the femoral head and the center of the sacral endplate in the cephalocaudal direction, and (3) increasing sacroiliac joint angulation. In Figure 1, the change in the PI from 30° to 50° to 80° corresponds to (1) an increased horizontal offset and (2) a decreased vertical pelvic offset. The association between a decreased vertical pelvic offset and an increased PI was also demonstrated by Bouly et al. in human cadaver studies [25]. These two factors of the PI increase could also explain the pathogenesis of variable PIs. In the case of subluxated hips, a superior shift of the femoral head may result in a decreased vertical pelvic offset, theoretically leading to a PI increase. Regarding (3), increasing sacroiliac joint angulation is often seen in cases of sacroiliac joint degeneration with adult spinal deformity. In cases of older patients with adult spinal deformity, the pelvis tilts backward to compensate for the decrease in lumbar lordosis or kyphosis. The excessive loading of a posterior pelvic tilt can induce the degeneration and instability of the sacroiliac joints. Furthermore, the trunk tilts forward, and the sacrum tilts forward, “increasing the angle of the sacroiliac joint” and resulting in a higher PI (Figure 2) [26].

## 4. The Relationship between the PI, Lumbar Disorder, and Sacral Morphology

### 4.1. The Relationship between the PI and Lumbar Disorder

Over the past 30 years, it has been thought that the PI may be related to certain spinal diseases [1,2,3]. Several studies have shown that, as the PI increases, more mechanical forces are transmitted to the lumbar spine [27,28]. In addition, it has been noted that an increased PI may be associated with a greater risk of developing or progressing to isometric spondylolisthesis, degenerative spondylolisthesis, scoliosis, accelerated disc degeneration, herniated discs, or facet joint arthritis [29,30,31,32,33,34,35]. In particular, an increased PI is associated with more sagittal facet joint angles and facet joint contact forces in the lower lumbar spine [35]. This actively demonstrates that these individuals primarily use their spines for the activities of daily living, which defines a flexible spine [2]. This may lead to the degeneration of the intervertebral discs and facet joints, which may be related to the development of degenerative spondylolisthesis or lumbar spondylolisthesis. Furthermore, Strube et al. [36] found a significantly lower PI in patients with degenerative disc disease compared to patients with lumbar spinal stenosis, lumbar disc herniation, and degenerative spondylolisthesis.

### 4.2. The Relationship between the PI and Sacral Morphology

Because sacroiliac joint mobility and degeneration are associated with an increased or decreased PI, the correlation of the PI with the sacroiliac joints and sacral morphology is also critical knowledge for understanding the pathophysiology of hip–spine syndrome. The anatomical studies of 120 cadavers have shown that a PI increase is associated with a greater sacral curve [37]. It has also been noted that a lumbar lordosis (LL) increase, which is associated with a PI increase, results in a greater sacral curve with more mobility of the sacroiliac joint. The mentioned anatomical facts contribute to the findings explaining the relationship between the degeneration and mobility of the sacroiliac joints and a PI increase.

## 5. PI–Hip Disorder Relationships

### 5.1. The Relationship between the PI and Hip Disorder

Table 1 summarizes 18 publications on the role of the PI in hip disease. All searches were conducted on 25 September 2022, and PubMed was utilized to search for relevant peer-reviewed articles published in the English language on the PI in patients with hip osteoarthritis.

The search term used in PubMed was as follows: (pelvic incidence [Title/Abstract]) AND (hip osteoarthritis [Title/Abstract]). Review articles, case reports (n < 3), commentaries, editorials, insights articles, conference abstracts, and proceedings were excluded. The references of the eligible studies were screened through a database search. This summary provides little evidence to support that the PI plays a substantial role in hip osteoarthritis (HOA).

By nationality, Japan had the highest number of reports (nine) followed by the USA (two), France (two), Canada (one), China(one), Turkey (one), Iran (one), and Poland (one). There were three prospective studies [38,41,50]. The oldest paper was reported in 2005 [47]; all the rest were reported after 2015, perhaps reflecting that most studies on this topic had just commenced. Notably, 9 of the 18 studies were conducted very recently within the limited post-2020 timeframe [43,44,45,46,50,51,52,53,54]. Nine of the non-Japanese reports were HOA reports [37,38,39,40,41,42,43,44,45], and two strictly excluded DDH [39,44]. Furthermore, it was noted that five out of the nine HOA reports from Japan contained DDH [48,49,50,52,55] because most HOA in the Japanese population is secondary to DDH [56]. Two studies focused on comparing HOA and RDC [48,54]. Five studies excluded hip dislocations classified into Crowe groups II, III, and IV [46,47,52] and Crowe groups III and IV [50,51]. The included studies varied in sample size from 25 to 1088 participants. The mean age was middle-aged or older, with participants in 4 studies being in their 50s, those in 11 studies being in their 60s, and those in 3 studies being in their 70s. Although the majority of the HOA patients reported in Japan were women, there was no sex predominance in the reports outside of Japan.

In this study, the mean PI of HOA showed more than 10° of variation (from 45.8 to 60.6), and the PI varied widely (from 18° to 96°) [33,38,39,40,41,42,43,44,45,46,47,48,49,50,51,52,53,54,55,56,57,58]. Similarly, in normal subjects, the PI ranged widely (from 35° to 85°) [6,7], and the lack of agreement on a “normal” reference PI value makes interpreting the results difficult [55].

Theoretically, greater PI values may cause a greater posterior pelvic tilt and a decreased anterior coverage of the femoral head at the acetabulum, which is associated with increased mechanical stress on the femoral head and may contribute to the development of HOA [25,47,49,57]. However, the relationship between the PI and HOA development has been controversial. Several studies argue that these two parameters are related [47,58,59], whereas others conclude that they are not [12,39,42,51]. Regarding the relationship between hip disease and the PI in past studies, the PI in HOA is a second-line research item and has not been fully explored. In addition, the pathogenesis of HOA is multifactorial, with predisposing genetic and local mechanical risk factors being involved. Therefore, stratification and analysis by HOA phenotype, such as femoroacetabular impingement (FAI), the development of the dysplasia of the hip (DDH), the osteonecrosis of the femoral head (ONFH), and rapidly destructive coxarthrosis (RDC), may help to characterize the PI.

### 5.2. The Relationship between the PI and FAI

Many studies support the finding that a low PI predisposes one to symptomatic FAI [2,12,58,60]. Riviere et al. [2] proposed the classification of the spinopelvic parameters as a risk factor for developing FAI. Patients with a low PI have a more marked cross-sectional anteversion of the acetabulum. Theoretically, these patients are more prone to femoral neck impingement syndrome because they are less able to adapt to sagittal plane imbalances with a tendency to hyperextend their hips [61]. In addition, a low PI pelvis may lose lumbar lordosis and require an anteversion of the pelvis to maintain sagittal balance, which may increase the symptoms of FAI [62].

### 5.3. The Relationship between the PI and DDH

**Figure 3:**
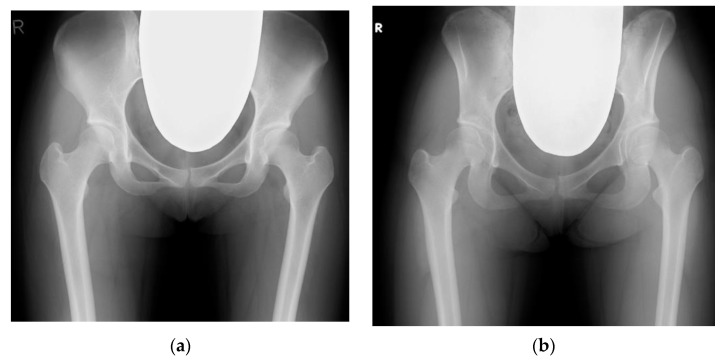
Development of dysplasia of the hip with varying severity. (**a**) Dysplasia of the acetabulum with pelvic incidence of 46°. (**b**) Dysplasia of the entire pelvis from the acetabulum to ilium (iliac wing curved inward) with pelvic incidence of 52°.

Despite the characteristic morphology of the pelvis and femur in patients with DDH [51], the role of the PI in DDH in the development of hip OA remains unknown [51,63]. In DDH, some reports indicate that the PI was significantly greater than that in the controls [64,65], while others suggest that they have no relationship [51]. In fact, Riviere et al. [2] proposed the classification of the spinopelvic parameters as risk factors for the development of FAI. With regard to DDH, Iwasa et al. [51] reported no significant difference in the PI between DDH and DDH-induced OA. However, the authors speculated that the mechanical stress in DDH could be significantly affected by an abnormal PI. However, there is diversity in the severity of DDH. In other words, DDH is not limited to the dysplasia of the acetabulum, and it includes the dysplasia of the entire pelvis from the acetabulum to the ilium (“iliac wing curved inward”) (Figure 3) [66]. The PI may vary depending on the type and severity of DDH dysplasia and requires more detailed studies.

### 5.4. The Relationship between the PI and ONFH and RDC

**Figure 4:**
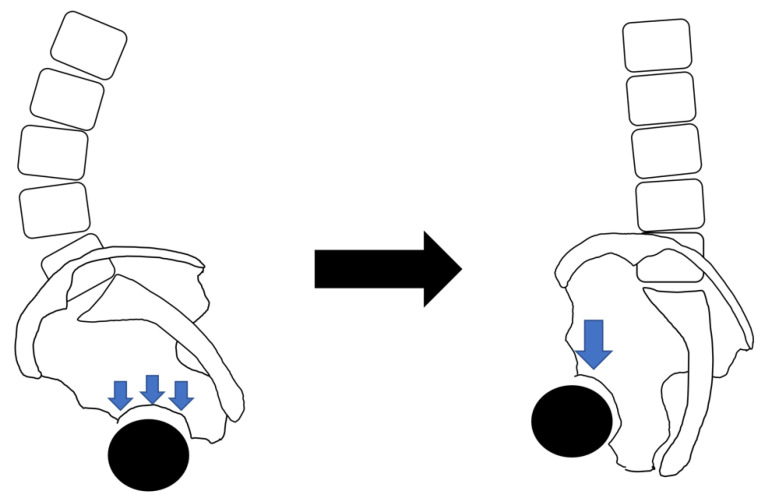
Stress concentration on the femoral head with greater PI, lumbar kyphosis, and posterior pelvic tilt. A case with greater PI, lumbar lordosis, and pelvic forward tilt (**left**) may have a relative decrease in anterior coverage of the acetabular head and increased abnormal stress on the head when the lumbar spine becomes kyphotic and when pelvis tilts posteriorly due to age-related degeneration (**right**).

There is a positive relationship between the PI and LL: the greater the PI, the greater the LL [2,25]. Due to age-related degeneration, decreased lumbar lordosis or lumbar kyphosis can cause a compensatory posterior pelvic tilt. Due to the larger PI and LL, the compensatory posterior pelvic tilt is greater when LL is reduced. A greater posterior pelvic tilt may decrease the anterior coverage of the femoral head at the acetabulum, which is associated with increased mechanical stress on the femoral head and may contribute to the development or worsening of several hip disorders (i.e., HOA, ONFH, and RDC) (Figure 4) [25,47,49,57]. Yoshimoto et al. [47] suggested that a greater PI and the resulting biomechanical adaptations in young people may contribute to the development of HOA later in life. A deeper understanding of the biomechanical adaptations that may result from an increased PI may be helpful in surgical planning and developing techniques for periprosthetic osteotomies and hip arthroplasties [57].

For ONFH, Kwon et al. [67] reported that a large PI is associated with the rapid development of ONFH and concluded that assessing the parameters in the pelvic sagittal plane in patients with early nontraumatic ONFH may help to predict which patients are at risk for a femoral head collapse. This finding may significantly impact how these patients are treated and followed up, and more confirmatory studies are needed [68].

RDC had a higher degree of femoral head destruction and more patients with severe pelvic discrepancies (PI–LL of >20° and PT of >30°in comparison to HOA) [49,55]. However, there was no significant correlation with the PI [55]. Because of the extremely low incidence of RDC, the relationship between RDC and the PI should be verified in a large prospective study.

### 5.5. What Is the Impact of HOA on the PI?

Conversely, it is necessary to consider whether HOA will change the PI. In advanced cases of HOA, the hip axis is shifted cranially due to the destruction and subluxation of the head. In other words, a vertical offset decrease occurs, resulting in a larger PI [8]. Therefore, although many reports clearly state that cases with a highly dislocated hip were not included, such cases should be excluded when investigating the PI in HOA. However, Okuzu et al. [69] reported that severe SIJ degeneration was often seen in patients with end-stage osteoarthritis secondary to unilateral DDH. This may result in increasing sacroiliac joint angulation [8] and may lead to a larger PI. In other words, HOA may increase. No studies on the PI in HOA cases considered the effect of sacroiliac joint angulation, which is one of the issues that will be addressed in future research.

## 6. Limitations and Future Directions

The present study has some limitations. First, as noted by Saltychev et al. [12], who examined the relationship between the PI and hip disease in a systematic review, omissions were common. In addition, concerning the study design in this review, most of the publications were retrospective studies. The relationship between the PI and HOA has not been fully explored because the PI in HOA was a second-line research item. Thus, the widely varying subjects, study designs, and methods did not allow for valid meta-analyses, nor could the methodological quality of the studies be systematically analyzed. Therefore, the lack of a systematic methodology in this review made it impossible to obtain the highest level of current evidence on the conditions and techniques. Another limitation of the narrative review was the inability to locate all the relevant literature or to cover the scientific literature without bias. Moreover, since half of the publications covered in this review were conducted as recently as after 2020, more data on this issue might emerge in the next few years, and the review may need to be updated fairly soon.

Second, we did not consider any important parameters of spinopelvic alignment other than the PI, specifically, lumbar lordosis, the pelvic tilt, the sacral slope in the sagittal plane, and lumbar scoliosis (i.e., the cobb angle) and pelvic obliquity in the coronal plane. Although correlations between these parameters and the PI have been reported, the distribution of these parameters varies among reports [2,43,49,52], which may result from genetic and environmental factors [52]. These are important factors to investigate to understand the relationship between HOA and spinal alignment, i.e., the pathophysiology of hip–spine syndrome, and they will be the subject of future studies. In addition, the PI was found to be associated with sagittal spinopelvic kinematics: a larger PI is related to higher lumbar flexibility and vice versa [52]. However, recent research has found an exception: some HOA patients have a lower PI, high lumbar lordosis, and high lumbar flexibility. Although the PI seems to be a leading predictor of sagittal spinopelvic kinematics, this exception may make it difficult to clarify the relationship between the PI and HOA.

Third, a deformity or dislocated hip and the degeneration of the sacroiliac joints, which affect the PI, have not been fully investigated. In HOA, the deformity or dislocation of the femoral head often results in a “decreased vertical pelvic shift,” which, theoretically, can increase the PI since the center of the femoral head (the hip axis) is often displaced in the cephalad direction. In addition, “increasing sacroiliac joint angulation” can increase the PI in cases of sacroiliac joint degeneration (Figure 2). The clarification and stratification of the subject will be important for future studies to understand the relationship between the PI and HOA.

Fourth, there were numerous reports from Japan. Several studies have reported that primary HOA is rare in the Japanese population and that it is mostly secondary to DDH, and racial differences in other countries have been noted [36,56]. In addition, anatomical morphology and the PI were reported to vary by ethnicity. Moreover, although the majority of the HOA patients reported in Japan were women, several studies comparing the PIs of women and men have shown no significant difference between the two groups [27,63].

Fifth, there may have existed a reporting bias, as some of the included studies [32,39] reported having financial conflicts of interest. Finally, since the subjects in this study were of middle or older age, and the PI is usually measured in the standing position, it is possible that their PIs were artificially high. Despite these limitations, our findings may help to further understand the relationship between the PI and HOA. They should be of interest to orthopedic surgeons and other healthcare professionals involved in musculoskeletal medicine.

## 7. Conclusions

The PI is the most important parameter for understanding the pathophysiology and mechanisms of hip–spine syndrome and for determining the treatment strategies for hip–spine syndrome. Because of the close relationship between the hip and the spine, orthopedic surgeons should always consider the spine when considering the hip and vice versa while simultaneously noting the PI.

An increased PI has also been observed in the evolution of bipedal locomotion in human evolution, in the acquisition of walking during child development, and in the standing posture of older individuals. While the PI may be associated with a greater risk for the development of or progression to spinal disorders, the association between the PI and hip disease remains controversial. It should also be noted that the cranial shift of the hip axis due to the destruction and subluxation of the femoral head with advanced HOA and sacroiliac joint angulation changes due to the accelerated degeneration of the sacroiliac joint with advanced HOA, theoretically, may increase the PI. Since the factors and phenotypes of HOA are diverse, more cases need to be investigated with stratification by HOA type before conclusions can be drawn on the relationship between HOA and the PI. Further investigation on this topic is therefore warranted.

## Figures and Tables

**Table 1:** Summary of the results.

Reference	Nationality	Inclusion	Number	Age; Mean ± SD (Range)	Sex; Men, Women	PI; Mean ± SD	PI; Range
[38]	France	HOA	50	64 (47–81)	26, 24	56.04	40–87
[39]	China	HOA	58	59.0 ± 11.9	N/A	49.0 ± 10.8	N/A
[40]	France	HOA	30	59.5 ± 15.6 (25–81)	12, 18	56.3 ± 11.5	35.1–81.4
[41]	Turkey	HOA	28	60.9 ± 7.3 (50–76)	11, 17	50	(35, 60) #
[42]	Iran	HOA	95	68 (43–93)	50, 45	56.0 ± 12.8	33–96
[43]	USA	HOA	1088	64.1 ± 10.4	557, 531	56.0 ± 11.5	N/A
[44]	Poland	HOA	50	N/A	N/A	50.9	19.7–78.4
[45]	USA	HOA	136	56.8 ± 10.8 (16–86)	77, 59	60.6 ± 12.0	N/A
[46]	Canada	HOA	52	63 ± 11 (38–82)	20, 32	57 ± 12	31–89
[47]	Japan	HOA	4	61.0 ± 11.1	30, 120	58.5 ± 14.0	29–90
		DDH	146				
[48]	Japan	HOA	64	65.5 ± 13.4 (27–86)	14, 60	54.7 ± 13.6	18–92
		ONFH	13				
[49]	Japan	HOA	70	75 ± 4	0, 70	55 ± 14	N/A
		RDC	44	79 ± 5	0, 44	58 ± 16	
[50]	Japan	HOA	77	62.2 ± 12.8 (21–84)	73	48.3 ± 15.8	18–70
[51]	Japan	DDH	100	65.5 ± 10.3	14, 86	53.7 ± 10.0	N/A
		HOA	100	61.0 ± 9.3	9, 91	52.3 ± 10.4	
[52]	Japan	HOA	945	64.4 ± 9.9 (34–89)	141, 804	53.3 ± 11.0	27–84
[53]	Japan	DDH	78	53.6 ± 12.9	21, 102	48.0 ± 8.9	N/A
		HOA	8				
		ONFH	10				
		Others (RDC, trauma)	6				
[54]	Japan	DDH	213	74.5 ± 7.0	30, 243	57.0 ± 13.9	N/A
		HOA	60				
[55]	Japan	HOA	25	71.9 ± 6.4	4, 21	45.8 ± 10.0	41.6–49.9
		RDC	34	74.6 ± 8.1	1, 33	49.6 ± 11.6	45.6–53.6

HOA represents hip osteoarthritis, DDH represents developmental dysplasia of the hip, RDC represents rapidly destructive coxarthrosis, ONFH represents osteonecrosis of the femoral head. # Median (interquartile range—25th, 75th percentile).

## Data Availability

Not applicable.
